# Response to Jors et al, *Environmental Health Insights*

**DOI:** 10.1177/1178630218788554

**Published:** 2018-07-20

**Authors:** Michael Eddleston

**Affiliations:** Centre for Pesticide Suicide Prevention, The University of Edinburgh, Edinburgh, UK

Dear Madam/Sir

We were pleased to see the editorial by Jors et al^1^ introducing a special issue on the major problems of pesticide poisoning facing low- and middle-income countries (LMICs). We were however disappointed to note the paucity of discussion on pesticide self-poisoning, the cause of most deaths from pesticide poisoning.

Pesticide self-poisoning is an occupational condition. If pesticides were not used occupationally in small-scale farming communities, they would not be available for self-harm. Self-poisoning would then be much less lethal (as it is in high-income countries where medicines are most typically taken in self-harm) and the appalling loss of life from pesticide suicide that accompanied the Green Revolution would not have occurred.^2^ It is a terrible shame that acute pesticide self-poisoning has been excluded from international treaties, such as the Rotterdam Convention. This has happened at great cost in human lives because it is blamed on the person’s action and not on the introduction of these highly hazardous pesticides into rural villages without the resources to use and store them safely.^3^

We also disagree strongly with the author’s statement that restricting access has been unsuccessful “largely because of failure to take account of adequate training and awareness about why locking up pesticides is vital” and with their citing our study^4^ in support.

Our trial was a large rigorous effectiveness cluster randomized controlled trial (RCT) that aimed to see whether well-designed household pesticide storage containers would prevent pesticide self-poisoning. It unfortunately clearly showed that such an approach is ineffective. The importance of storing pesticides safely was emphasized to householders when the containers were handed over, at 2 weeks when we checked that they had been installed in the ground, and at 6-monthly reminders to intervention communities. Effective use of containers might require frequent and sustained reminders to householders; however, in our RCT, we did not see any evidence that the approach worked in the first few months when use of containers will have been at its highest.

The belief over the last 10 to 15 years that “safe storage” should work, and the choice to blame suicide on pesticide misuse, thereby removing it from international conventions, has put suicide prevention in LMIC back several years and contributed to a large number of unnecessary deaths. It is time that the global community accepts pesticide self-poisoning as a complication of occupational pesticide use and supports pesticide regulation to completely remove highly hazardous pesticides from poor rural communities. As has been shown in multiple countries, this approach prevents all forms of acute pesticide poisoning and rapidly reduces total suicides.^5^
